# Supplementary material for: Body odor disgust sensitivity (BODS) is related to extreme odor valence perception
Source: PLoS One. 2023 Apr 21;18(4):e0284397. doi: 10.1371/journal.pone.0284397 (PMC10120931; doi:10.1371/journal.pone.0284397)

Body odor disgust sensitivity (BODS) is related to extreme odor valence perception: Supplementary materials

1. Evaluation of BODS-valence relationship in individual studies

In order to make sure the relationship between BODS and valence ratings is not driven by one of the four studies in particular, we took the best model from our main analysis (including an interaction between odor category and external subscale of BODS) and ran in separately for each study. The estimated effects were quite consistent across the studies, as illustrated in Figure S1 below.

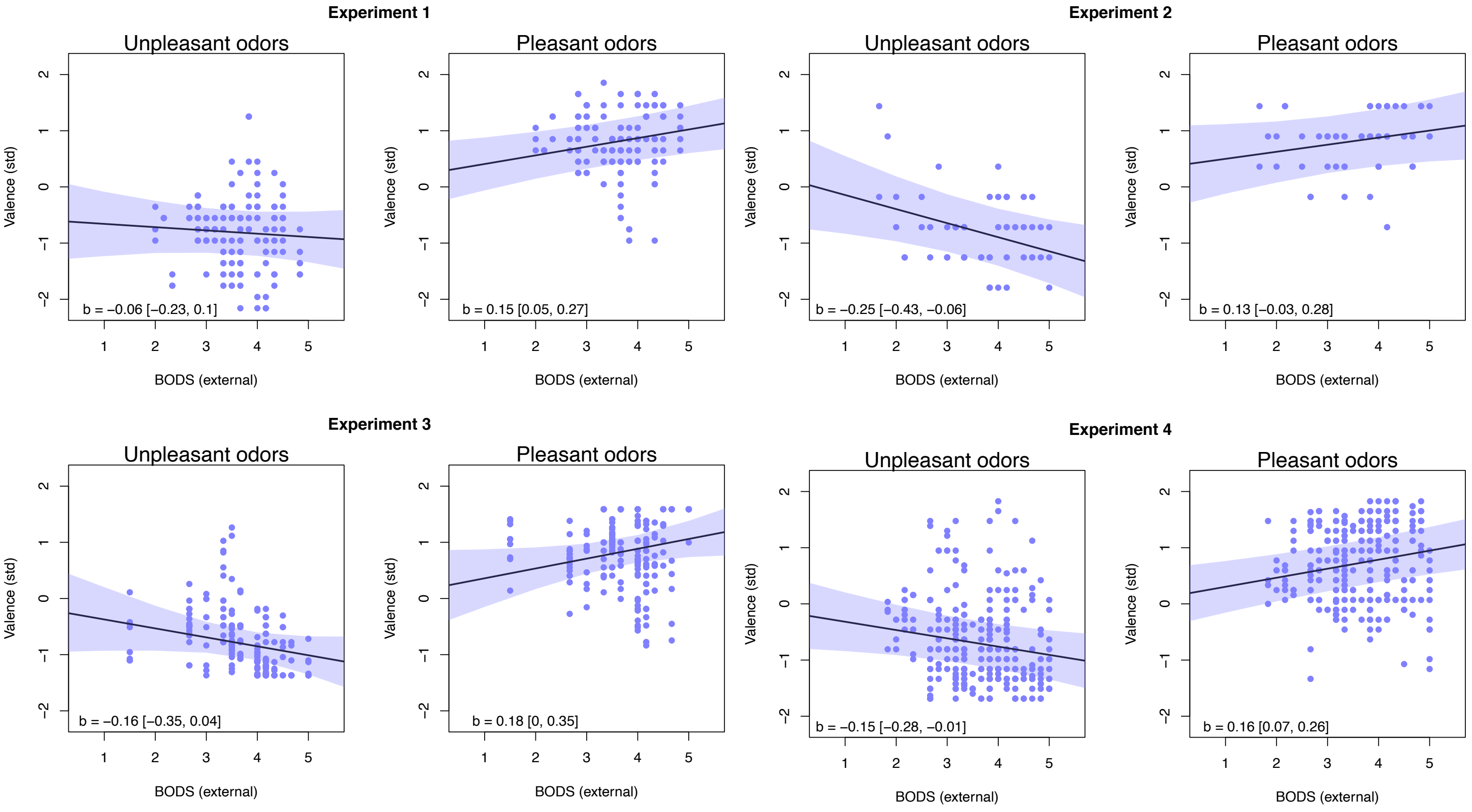

Figure S1. Relationship between external BODS and standardized valence ratings for unpleasant (left) and pleasant (right) odors. The data is obtained using the model that was best at predicting valence when using the entire dataset. The shaded area and numbers in square brackets represent 94% HPDI for the slope.

2. Exploratory analysis of gender differences in odor ratings

As an additional step, we investigated gender differences in the effects that odor category and BODS have on valence and intensity ratings. We were interested to see if the observed gender differences in odor ratings apply to both pleasant and unpleasant odors. We also wanted to investigate if the effects of BODS on valence (positive for pleasant odors, negative for unpleasant odors) are similar for the two genders. To do this, we took the best models from step 1 of our analysis (in which we used the overall BODS score) for both valence and intensity and added relevant interaction terms. We refer to these best models from step 1 as *original models*.

2.1. Gender differences, body odor disgust sensitivity and valence ratings

For valence, we first created three new models that extended the original model by: 1) a gender and odor category interaction, 2) a gender and BODS interaction, 3) both of these interactions. Next, we created models that included even a three-way interaction between gender, odor category and BODS. We made four new extensions of the original model with 1) the three-way interaction, 2) the three-way interaction, and an interaction between BODS and odor category, 3) the three-way interaction, and the interaction between gender and BODS, 4) the three-way interaction and both two-way interactions (gender and BODS, gender and odor category). To sum up, we had seven new models of valence ratings, with increased complexity, capturing the possible joint effects of our variables of interest (gender, odor category and BODS).

We compared all the new, extended models with the original model. As in the main analysis, for a model to be considered better, it had to have a lower WAIC value than the alternative models, and this difference had to be at least twice as big as the ΔSE (ΔWAIC /Δ SE >= 2). If models were not distinguishable based on WAIC, the simplest model was considered best. To support the understanding of this complex model comparison we also provide information from comparing a subset of models in a one-to-one fashion, including a direct comparison of all new models with the original model.

2.1.1. Women rate (pleasant?) odors as more pleasant

None of the extended models fit the data better than the original model (ΔWAIC /Δ SE < 2, Table S1). To better understand this complex comparison output, we show also results for a direct one-to-one comparison of the original model with each of the extended models (Table S2). Models with the three-way interaction had lowest WAIC values and were almost indistinguishable (ΔWAIC < 1). They were not substantially better than the simpler models without a three-way interaction (ΔWAIC /Δ SE < 2). Although the difference between the top three-way interaction model and the original model was only slightly below our criteria (ΔWAIC /Δ SE = 1.8), the additional complexity of the model is hard to be justified.

For exploratory purposes, we present predictions from one of the simpler models including the interaction between gender and odor category (Figure S2). We chose to present results from this model, as it might be chosen if we were to have less conservative WAIC difference criteria, and has a better complexity vs. fit to data trade-off. It fits the data similarly to the three-way interaction model with lowest WAIC value (ΔWAIC = 4; ΔWAIC /Δ SE = 1.8), yet has a simpler interaction term. The gender and odor interaction suggests that women perceive smells as more pleasant (as in the original model), however, this is the case only for pleasant and not unpleasant odors (Figure S3).

For a curious reader, we illustrate even the predicted results of the lowest WAIC model (top row in Table S1, model that included the effect of BODS and odor category interaction from the original model and the three-way interaction between gender, odor category and BODS) on Figure S3.

Table S1. Model comparison for models predicting valence ratings, extended by adding gender interactions. Models are presented based on their WAIC value: lowest (i.e., better; top row) to highest (i.e., worse; bottom row). WAIC - widely applicable information criterion; Δ WAIC – WAIC difference (vs. model in the top row); Δ SE - standard error of the WAIC difference. BODS overall – body odor disgust sensitivity overall score. The original model included an interaction between odor category and BODS, the main effect of gender and random intercepts for each participant. * Indicates an interaction effect and + an additive effect.

(Odor + BODS + Odor * BODS)	**WAIC**	**ΔWAIC**	**Δ SE**	**ΔWAIC /Δ SE**
+				
Gender * Odor * BODS	2657	-	-	-
Gender * Odor * BODS + Gender * BODS	2657	0.0	1.5	0.0
Gender * Odor * BODS + Gender * Odor	2657	0.6	0.7	0.9
Gender * Odor * BODS + Gender * Odor + Gender * BODS +	2658	1.0	1.6	0.6
Gender * Odor + Gender * BODS	2660	3.2	2.7	1.2
Gender * Odor	2661	4.1	2.3	1.8
Gender * BODS	2673	16.1	9.7	1.7
(original model)	2675	17.8	9.8	1.8

Figure S2. Valence ratings for men (blue diamonds) and women (gray circles) for pleasant (left) and unpleasant (right) odors. The density of the data is additionally illustrated by the intensity of the color. Black horizontal lines reflect mean ratings as predicted by the gender and odor category interaction model, dashed lines and the shaded area reflect 94% HPDI.

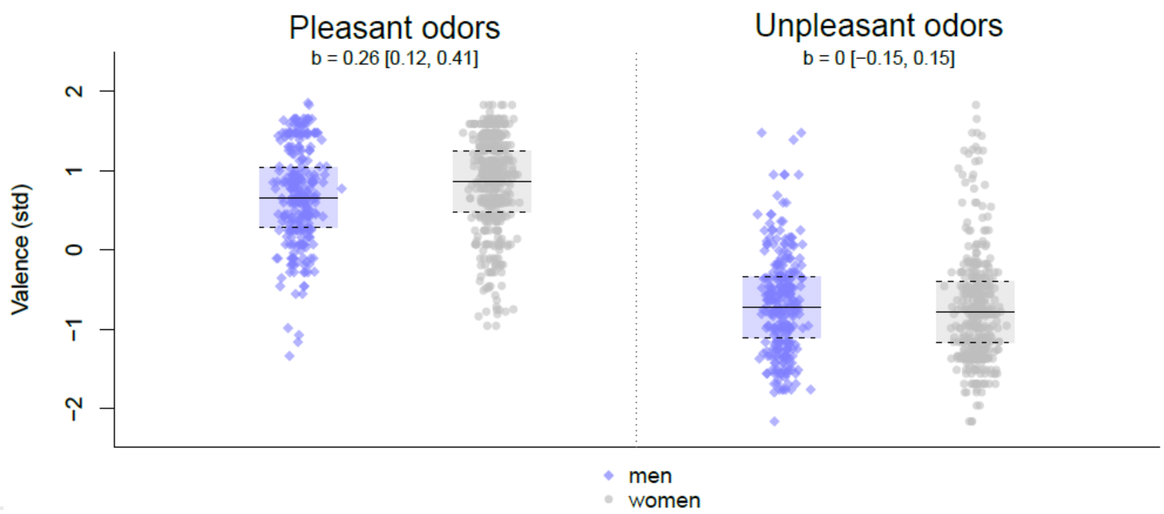

Table S2. Model comparison for the extended models of valence ratings versus the original model (best model in step 1 of data analysis). The original model is on top of the table, followed by remaining models that presented in order of increased complexity. Negative values indicate that the model had lower (better) WAIC than the original model. WAIC - widely applicable information criterion; Δ WAIC – WAIC difference (vs. model in the top row); Δ SE - standard error of the WAIC difference. BODS overall – body odor disgust sensitivity overall score. The original model included an interaction between odor category and BODS, the main effect of gender and random intercepts for each participant. * Indicates an interaction effect and + an additive effect.

	WAIC	ΔWAIC	Δ SE	ΔWAIC /Δ SE
Odor + BODS + Odor * BODS (original model)	2675	-	-	-
+				
Gender * Odor	2661	-13.7	9.2	1.5
Gender * BODS	2673	-1.7	2.2	0.8
Gender * Odor + Gender * BODS	2660	-14.6	9.1	1.6
Gender * Odor * BODS	2658	-16.9	9.6	1.8
Gender * Odor * BODS + Gender * BODS	2657	-17.8	9.5	1.9
Gender * Odor * BODS + Gender *Odor	2657	-17.2	9.7	1.8
Gender * Odor*BODS + Gender * Odor + Gender * BODS	2658	-16.9	9.6	1.8

Figure S3. Relationship between BODS and valence ratings for men (blue diamonds) and women (gray circles) for pleasant (left) and unpleasant (right) odors. The density of the data is additionally illustrated by the intensity of the color. Horizontal lines reflect relationship between BODS and valence predicted by the three-way gender, odor category and BODS interaction model for women (gray) and men (blue). Shaded areas reflect 94% HPDI.

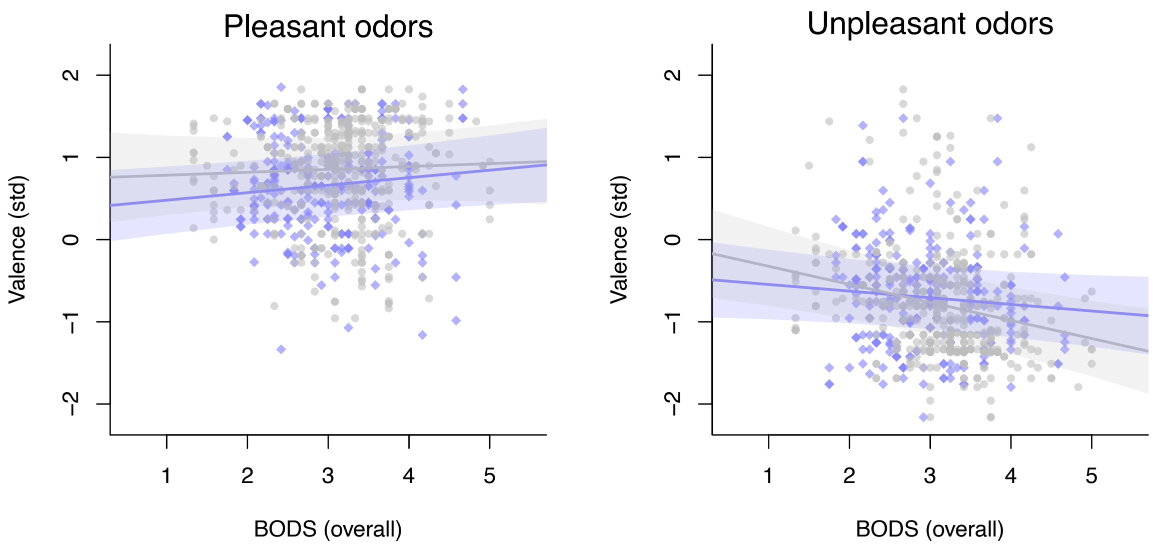

Gender differences in intensity ratings

For intensity, we extended the best model by adding a gender and odor category interaction (as the best model for intensity did not include BODS). We then compared this new model to the original based on the same WAIC criteria described above.

2.2.1. Men rate odors as somewhat less intense than women

The model with gender and odor category had a slightly higher WAIC value than the original model (ΔWAIC = 2.2, Table S3), and the simpler, original model should be considered best. Fore exploratory purposes, we illustrate predictions from the interaction model in Figure S4. Model comparison for models predicting intensity ratings, extended by adding gender interaction. Models are presented based on their WAIC value: lowest (i.e., better; top row) to highest (i.e., worse; bottom row), and the best model is marked in bold. WAIC - widely applicable information criterion; Δ WAIC – WAIC difference (vs. model in the top row); Δ SE - standard error of the WAIC difference. The original model included the effect of odor category, main effect of gender and random intercepts for each participant. * Indicates an interaction effect and + an additive effect.

	**WAIC**	**ΔWAIC**	**Δ SE**	**ΔWAIC /Δ SE**
**Odor (original model)**	**3709**	**-**	**-**	**-**
Odor + gender * Odor	3711	2.2	3.8	0.6

Figure S4. Intensity ratings for men (blue diamonds) and women (gray circles) for pleasant (left) and unpleasant (right) odors. The density of the data is additionally illustrated by the

intensity of the color. Black horizontal lines reflect mean ratings as predicted by the gender and odor category interaction model, and dashed lines and the shaded area reflect 94% HPDI.

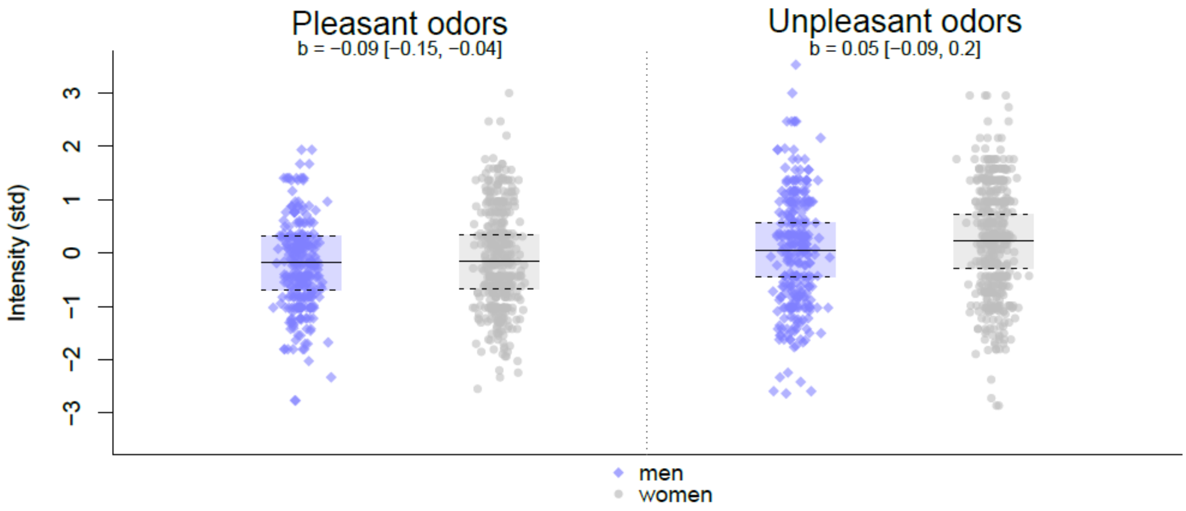

Supplement: S1 Appendix — (PDF) [file pone.0284397.s001.pdf]
