# Supplementary material for: Body odor disgust sensitivity (BODS) is related to extreme odor valence perception
Source: PLoS One. 2023 Apr 21;18(4):e0284397. doi: 10.1371/journal.pone.0284397 (PMC10120931; doi:10.1371/journal.pone.0284397)
Supplement: Attachment — Submitted filename: Response to Reviewers.docx [file pone.0284397.s002.docx]

*Dear Editor,*

*We would like to thank the Editor and the Reviewers for their work and insightful comments and suggestions. We did our best to answer each question and incorporate the suggestions into the manuscript. Below, we present the original comments/questions in regular font, each point marked by an index in bold (from* **R1.1** *to* **R2.14***), followed by our answer in italics.*

*We would like to point out that in the updated version of the manuscript, there are now additional tables and the results section has been restructured; therefore, table and figure numbering is different to the previously submitted file. The new submission includes also a short supplementary materials document. We tried to make the text clearer and changed the wording in several places where we thought we had not done our best in the first submission.*

*We hope that the Editor and both Reviewers will find the changes satisfactory. Please let us know if any additional adjustments are required or if anything is not up to your expectations.*

*Best regards,*

*Authors*

*Response to Reviewers’ comments:*

**Reviewer #1:** 

**R1.1.** The aggregated data came from experiments that applied quite different methodology, in particular on odor presentation. This may have affected the related ratings. How the authors dealt with these possible differences? Did the authors perform a comparison of the standardized data between experiments? If there is a difference, could it have affected final results? Please clarify

*Thank you for this comment. We interpret it as referring to either a request for the correlation results, or the odor ratings differences, pertaining to the 4 individual studies. In terms of the regression slopes, they are highly consistent across studies (see figure R1 below, or Figure S1 in Supplementary Materials). In terms of the odor ratings, they are relatively consistent in terms of valence, but they differ more in terms of intensity, as can be expected given the variations in odor presentation (see Table 3 in the manuscript). Please have a look at what we wrote on a similar comment from Reviewer 2 (point R2.6), as well as Figures R2 and R3*. *We regard our results as being strengthened by the fact that similar effects are obtained across studies, despite differences in methodology. We hope that these data and results address the reviewer comment.*

*Figure R1. Relationship between external BODS and standardized valence ratings for unpleasant (left) and pleasant (right) odors. The data is obtained using the model that was best at predicting valence when using the entire dataset. Shaded area and numbers in square brackets represent 94% HPDI for the slope.*

**R1.2.** Line 157: “Perceptual ratings were done before and after the task.” In this case, which ratings were included in the analyses? Only one data per participant, an average of the repetition or multiple data?

*For all experiments, we used all ratings just as they were given, as separate data points in the analysis, without averaging. Number of ratings done by each participant for each odor is indicated in Table 2 in the Trials per odor column. Thus, in case of experiment 2 to which the cited sentence refers to, both pre and post ratings were used in the model without averaging, included as two separate data points from every participant. The dependency of ratings given by the same person is taken into account, since we used participant ID as a random effect in the model. This is additionally clarified in the manuscript (page 7, lines 212-214)*

*“As each individual in the dataset gave multiple ratings of each odor, all models allowed individual intercepts for overall ratings for each participant (random effect of participants ID).”*

**R1.3.** Please include a table of Means/SD of all key variables (pleasantness intensity ratings of each odor, BODS) per experiment

*Thank you for this great suggestion. We added two new tables in the revised manuscript: Table 1 now includes demographic information (sample size, age, moved from Table 2) and descriptives for BODS scale in each study; Table 3 shows pleasantness and intensity ratings for pleasant and unpleasant odors (on raw scales) in each experiment.*

**R1.4.** In general, the discussion is very long and quite difficult to follow. I suggest the author simplify some parts. For example, the paragraph starting at line 384 is very long and not well connected with the rest of the discussion.

*Thank you for this feedback. We have shortened the discussion, specifically the paragraph mentioned, which is now only one sentence long (page 13, lines 419-421).*

**R1.5**. Line 384 “Our converge with those of a recent study by Tybur and colleagues”: I think this sentence is missing a word

*The sentence is now corrected.*

**R1.6.** Line 404 “Our perceptual rating results suggest that people with higher BODS levels are more likely to have a lower criterion i.e., less is needed for them to judge an odor as unpleasant or pleasant, rather than then suggesting that they detect the odor more easily...” This sentence is unclear

*We have now changed the sentence mentioned to (page 13, lines 426-429): “As levels of BODS affected valence ratings, but not intensity ratings, we hypothesize that people with higher BODS levels are more likely to have a lower criterion: they more easily judge an unpleasant odor as unpleasant (or pleasant odor as pleasant), rather than detect the odor itself more easily”*

**Reviewer #2:**

**R2.1.** The authors describe the negative odors as “pathogen-related cues” and the positive odors as “health-related”. While the pathogen association seems quite strong based on the stimuli chosen, the positive/health related connection is still rather indirect and feels more like speculation; e.g., “lemon” could be associated with a lot of non-health related concepts too. Is there a previous study which validates this connection for the current stimuli?

*We have revised the description of pleasant odors:*

*- in the abstract (page 1, lines 22-23): “The pleasant odors were, in contrast, often found in soap and cleaning products (e.g., lilac, lemon).”*

*- introduction (page 2, lines 70-72) to: “...both pleasant (e.g., safety, cleanness or health–related) and unpleasant (e.g., potentially threatening, disease-relevant) odors. We included odors that might resemble or be associated with ‘real’ body odors, or fragrant products used on the body”*

*- and in the methods (page 5, lines 159-161): “Pleasant stimuli included odors associated with food, flowers and cleaning products, and thus assumed to have positive health connotations: lilac, lemon, peppermint and lily of the valley essences. Lilac in particular has been previously linked to cleanness (Syrjänen et al., 2019).”*

**R2.2.** The results reported show relatively small effect sizes; the model for valence ratings including BODS barely passes the 2 SE criterion compared to the Odor model. The interpretation and discussion of the results should reflect the size of the result more. A single sentence at the end of the conclusion does not quite acknowledge this.

*We agree with the reviewer that the effects we find are modest in size; for example, with 1 SD increase in overall BODS score the pleasantness ratings decrease about -0.11 SD for unpleasant odors, or increase by 0.16 SD for pleasant odors. We now mention this in the abstract of the paper (page 1 line 30):*

*“The effects were modest in size.”*

*However, the differences in information criteria (WAIC) is not a measure of effect sizes, they merely reflect which model does better at predicting the data. Although some models are barely above (or below) the 2SE criterion, this threshold should be considered conservative. A common practice is to choose a model based solely on difference in the WAIC value, without taking into account the standard error of the difference.*

*We would like to also mention that when using multilevel modeling, the interpretation of effect sizes changes somewhat, and that the effects are typically smaller. This is related to the fact that by using these models, we partial out some of the other sources of variability (see e.g., Brysbaert, 2019; Judd et al., 2017).*

Brysbaert, M. (2019). How Many Participants Do We Have to Include in Properly Powered Experiments? A Tutorial of Power Analysis with Reference Tables. *Journal of Cognition*, *2*(1), 16. https://doi.org/10.5334/joc.72

Judd, C. M., Westfall, J., & Kenny, D. A. (2017). Experiments with More Than One Random Factor: Designs, Analytic Models, and Statistical Power. *Annual Review of Psychology*, *68*(1), 601–625. https://doi.org/10.1146/annurev-psych-122414-033702

**R2.2a.** As an additional note to this. In some following comments I question whether certain additional factors should be considered (e.g., gender interaction and study number). Since the results are so small it becomes imperative to show that these factors do not bias the results.

*We understand the concern, but we already present evidence from 4 different empirical studies, which differ in some methodological aspects, and we apply a statistical model that was determined a priori. We find highly consistent results, as is now clarified. We had no reason for adding gender interactions, for reasons that are outlined below (point R2.4). As the data is standardized within each study, adding the study as a factor in the model is not warranted. In fact, modeling a different intercept for study number would be inappropriate for our data, as we forced the mean value for each study to be the same (zero) by standardizing the data. If the results were not robust, they would not have been close to identical across 4 separate experiments.*

**R2.3.** As a general note, the manuscript seems to focus more on model comparison than on the contents of the models. While I personally learned a lot about Bayesian parameter estimation by reviewing this manuscript, I assume that the connection between BODS and valence ratings is the take-home message here. Reporting all model comparisons in a single separate paragraph in the results would allow the authors to stress their findings more in the other sections.

*We have now changed the structure of the results section and we hope the important findings are highlighted in a better way.*

Major comments:
**R2.4** Line 200: Was a potential interaction of gender with the other factors explored? In addition to gender main effects, previous studies have shown that the influence of some factors on olfactory perception depends on the participant’s gender. These potential interactions should be addressed and tested for.

*Thank you for this comment. The reason we include gender in our models is because we perceive it as a potential confounder: it is a variable that (based on existing research) potentially influences both our outcome (odor perception) and the predictor of interest (disgust sensitivity). We did not hypothesize a different effect of gender on olfactory perception in the different BODS levels, nor a different effect of BODS on olfactory perception in the two gender groups. For this reason, we did not include an interaction model. As for odor category, we are not aware of studies showing that gender influences odor perception specifically based on the odor valence. Both questions would be worth exploring in a new study with specific, well-justified hypotheses about the relationships/interactions. We believe, however, that they are beyond the scope of the current paper.*

**R2.5** Line 264: Please describe the standardization process and its validity for comparing the different scales used in the studies. Did the data obtained in the different studies present similar distributions?

*We used a universal standardization process in which every single rating was first centered (meaning that the mean of all ratings in a given study was subtracted from the single rating) and then divided by the standard deviation (SD) of all ratings in the given study. As a result, the data from each experiment have a mean of zero, and the data points reflected deviations from the mean rating in the experiment, expressed in SDs. Thus, we obtained a unified unit (SD) for all experiments. We have added a table (Table 3) showing the ratings from each experiment before standardization, so that readers have a better idea of how similar (or different) the valence and intensity ratings were across experiments. To ease comparisons, we also converted the raw scores into proportion of max on the scale used. Odor ratings were relatively consistent in terms of valence, but they differ more in terms of intensity, as can be expected given the variations in odor presentation. Figures R2 and R3 below show the distribution of valence and intensity ratings separately for pleasant and unpleasant odors in each experiment.*

*Figure R2. Density plot for standardized valence ratings in each experiment for pleasant (blue) and unpleasant (gray) odors EXP = Experiment.*

*Figure R3. Density plot for standardized intensity ratings in each experiment for pleasant (blue) and unpleasant (gray) odors; EXP = Experiment.*

**R2.6** On a related note: Was the study in which each data point was obtained tested as a potential confound? Especially the different stimulation methods used in the studies could bias results. Additionally, the different stimulus presentation techniques should be listed as a limitation of the study.

*Thank you for this comment. This is our reasoning: if there are effects of the study or stimuli delivery method on ratings, it is safe to assume that this effect would be similar for all participants (i.e., it would be treated statistically as a between-subject experimental condition). Thus, if the data is standardized within an experiment (reflecting how each rating is in relation to the mean rating in a given study, expressed in standard deviations), we believe potential differences in baseline levels for intensity and/or valence ratings are not affecting our results. As described in an answer to Reviewer 1’s comment (point R1.1), the results are quite consistent across the four experiments (Figure R1). In our opinion, the fact that similar effects are obtained across studies, despite differences in methodology is a strength of the study rather than a limitation.*

**R2.7** Line 268: The formatting of the results section makes the manuscript difficult to navigate: Both sections 3.1 and 3.2 contain just a single sub-paragraph, questioning the point of using sub-paragraphs. The sub-title for 3.2.1 can simply be removed.
Section 3.3 discusses odor valence ratings, which is the title of section 3.1. The text would be easier to follow if this was moved up and described as section 3.1.2, which would also validate the use of a sub-paragraph for 3.1.1

*We agree that the result section would benefit from restructuring. We have now grouped the results relating valence to follow each other directly, and report intensity results at the end. We also added two paragraphs about mean raw valence ratings, and mean raw intensity ratings (including new tables with ratings in each study before standardization) to the corresponding sections. Hopefully this also makes the sub sections more justified and better organized.*

**R2.8** Tables 2/3/4: Due to their format, these can be difficult to interpret: Rather than looking at a model and checking whether alternatives are better, the reader has to find the best model and verify that the alternatives are worse (WAIC/SE values are shown in the form of increases rather than decreases). To improve readability I suggest highlighting the best fitting model in each of the tables in some way (e.g. bold/italics).

*We have highlighted the best models in each Table using bold font and added the following text to table captions: “Models are presented with regard to their WAIC value: lowest (i.e., better; top row) to highest (i.e., worse; bottom row), and the best model is marked in bold”*

**R2.9** Line 335: Description of table 4 refers to a), b), and c), though this summation isn’t used as such in the table. I suggest simply removing the letters. Arguably, the entire specification could be left out since stating that “BODS internal” refers to the “internal subscale of the BODS” seems a bit unnecessary.

*We have removed a repetition occurring in the Table caption: “score on the internal subscale (BODS internal) – score on the internal subscale” is now changed to “score on the internal subscale (BODS internal)”. We also followed the suggestion to remove the a), b), c) lettering.*

**R2.10** Line 176: Including a citation in a header feels odd. Adding it to the first mention of the questionnaire in the paragraph improves readability.

*This is now corrected.*

**R2.11** Line 397: Random period mid-sentence.

*This is now corrected.*

**R2.12** Line 401/402: Phrasing of the parameters is inconsistent. I suggest changing either the first to “how sensitive are they to…” or the second to “what their criterion is....”

*Thank you for this very good point, we have changed the phrasing now to (page 13, lines 425-426): “…sensitivity/easiness to detect a disgusting odor (i.e., the detection threshold, referred to as d’) and sensitivity/easiness to call an odor disgusting (i.e., the criterion).”*

**R2.13** Line 416: Since odor detection threshold was not measured, wouldn’t this make the “conclusion” regarding this point a “hypothesis” instead?

*We rephrased the sentence mentioned and incorporated this valid suggestion (page 13, lines 426-429).*

**R2.14** Figures: The figure captions don't describe the contents of the image sufficiently. What does "Shaded area and numbers in square" mean?

*Thank you for pointing this out. We did not notice that the captions were cut in the compiled pdf file. As it appears the same in the revised submission and seem to be beyond our control (full text is entered when submitting the figures), we provide the full captions here:*

*Figure 1. Relationship between overall BODS score and standardized valence ratings for unpleasant (left) and pleasant (right) odors. The shaded area and numbers in square brackets represent 94% HPDI for the slope.*

*Figure 2. Relationship between external BODS and standardized valence ratings for unpleasant (left) and pleasant (right) odors. The shaded area and numbers in square brackets represent 94% HPDI for the slope.*

*Figure 3. Standardized valence (left) and intensity (right) ratings for pleasant (white) and unpleasant (blue) odors. Black lines show medians, numbers in square brackets represent 94% HPDI for mean posterior estimate of the difference (b).*
