# Supplementary material for: Body odor disgust sensitivity (BODS) is related to extreme odor valence perception
Source: PLoS One. 2023 Apr 21;18(4):e0284397. doi: 10.1371/journal.pone.0284397 (PMC10120931; doi:10.1371/journal.pone.0284397)
Supplement: Attachment — Submitted filename: Respone to reviewers.docx [file pone.0284397.s003.docx]

*Dear Editor,*

*Again, we would like to thank the Editor and the Reviewers for their work and insightful comments and suggestions. Below we provide a summary of changes in this new version of our manuscript.*

*Best regards,*

*Authors*

*Response to Reviewers’ comments:*

1. We have addressed the final concern raised by Reviewer #2.

We realized that the Reviewer has a good point it highlighting that ‘*verifying whether [the interaction] affects the optimal model is important*’. We performed additional analyses in which we investigated potential gender differences in the effects of BODS on valence ratings (also looking separately at pleasant and unpleasant odors), as well as any potential interactions between gender, BODS and odor category. Similarly, we looked into gender differences in intensity ratings. These exploratory analyses and their results are brought up in the main text and described in detail in *Supplementary materials*. We hope that our analysis help clarify the potential differences between men and women.

Relevant text in the main manuscript:

- Methods section: *2.5.3 Exploratory analysis of gender differences* (p. 8 lines 288 – 291)
- Results: “*Exploratory analysis suggests that this gender difference might apply mostly to pleasant odors (see Supplementary materials, 2.1.	Gender differences, body odor disgust sensitivity and valence ratings)”*(p. 10, lines 358 – 360)
- Results: “*The exploratory analysis did not provide any more insight into gender differences (see Supplementary materials, 2.2. Gender differences in intensity ratings)”* p.11 lines 415 – 417)

2. We did minor updates to the text itself in order to correct previously unseen linguistic mistakes, and to improve the reading experience.
3. We realized we made a mistake when describing gender differences in intensity ratings in the previous submission, saying that it was women who rated smell as somewhat less intense, while in fact, it was men who rates smells as less intense than women. This effect is correctly reported in the current submission. (p. 11, line 416)
